# Spatial Clustering of Dengue Fever Incidence and Its Association with Surrounding Greenness

**DOI:** 10.3390/ijerph15091869

**Published:** 2018-08-29

**Authors:** Chi-Chieh Huang, Tuen Yee Tiffany Tam, Yinq-Rong Chern, Shih-Chun Candice Lung, Nai-Tzu Chen, Chih-Da Wu

**Affiliations:** 1 Department of Forestry and Natural Resources, National Chiayi University, Chiayi 60004, Taiwan; wsxqaza12@gmail.com (C.-C.H.); tiffanytam0809@gmail.com (T.Y.T.T.); s1040109@mail.ncyu.edu.tw (Y.-R.C.); 2 Research Center for Environmental Changes, Academia Sinica, Taipei 11529, Taiwan; sclung@gate.sinica.edu.tw; 3 Department of Atmospheric Sciences, National Taiwan University, Taipei 10617, Taiwan; 4 Institute of Environmental Health, National Taiwan University, Taipei 10055, Taiwan; 5 National Institute of Environmental Health Sciences, National Health Research Institutes, Miaoli 35053, Taiwan; cntlichi@nhri.org.tw; 6 Department of Geomatics, National Cheng Kung University, Tainan 70101, Taiwan

**Keywords:** dengue fever, surrounding greenness, spatial clustering, GLMM

## Abstract

With more than 58,000 cases reported by the country’s Centers for Disease Control, the dengue outbreaks from 2014 to 2015 seriously impacted the southern part of Taiwan. This study aims to assess the spatial autocorrelation of the dengue fever (DF) outbreak in southern Taiwan in 2014 and 2015, and to further understand the effects of green space (such as forests, farms, grass, and parks) allocation on DF. In this study, two different greenness indexes were used. The first green metric, the normalized difference vegetation index (NDVI), was provided by the long-term NASA MODIS satellite NDVI database, which quantifies and represents the overall vegetation greenness. The latest 2013 land use survey GIS database completed by the National Land Surveying and Mapping Center was obtained to access another green metric, green land use in Taiwan. We first used Spearman’s rho to find out the relationship between DF and green space, and then three spatial autocorrelation methods, including Global Moran’s I, high/low clustering, and Hot Spot were employed to assess the spatial autocorrelation of DF outbreak. In considering the impact of social and environmental factors in DF, we used generalized linear mixed models (GLMM) to further clarify the relationship between different types of green land use and dengue cases. Results of spatial autocorrelation analysis showed a high aggregation of dengue epidemic in southern Taiwan, and the metropolitan areas were the main hotspots. Results of correlation analysis and GLMM showed a positive correlation between parks and dengue fever, and the other five green space metrics and land types revealed a negative association with DF. Our findings may be an important asset for improving surveillance and control interventions for dengue.

## 1. Introduction

Dengue fever (DF) threatens the health of populations around the world because of the expanding distribution of the virus and its mosquito vectors over the past 30 years [1,2,3,4]. Dengue is a prominent mosquito-transmitted viral disease in humans that is a significant public health consideration in tropical and subtropical regions of the world in which it is endemic [5,6,7]. The true impact of dengue globally might be underestimated due to inadequate disease surveillance, misdiagnosis, and low levels of reporting [7,8,9]. The result of a statistical study has shown that 3.9 billion people, in 128 countries, are at risk of viral dengue infection, and more than 390 million cases are reported annually, which also includes Taiwan [3,10].

Dengue outbreaks have occurred every year in Taiwan since 1997, and the constant importation of cases of the virus from dengue-endemic countries is responsible for the local outbreaks each year [11,12,13]. Moreover, southern Taiwan’s ecological environment is very suitable for the breeding of both *Aedes aegypti* and *Aedes albopictus* [12,14], which makes it easy to form the human-to-mosquito-to-human cycle of transmission of DF. In recent years, the dengue epidemic has risen sharply in southern Taiwan. A total of more than 58,000 cases were reported from 2014 to 2015, which is the highest in Taiwan in the past decade. Dengue currently represents an important vector-borne disease in Taiwan.

The environmental characteristics and the occurrence and dissemination of disease are inseparable [15,16]. In previous studies, climatic conditions, including temperature, precipitation, and humidity, are the major drivers which have been highlighted. In addition to climate drivers, land use and sociocultural practices also have strong relationships with dengue incidence [17,18]. In recent years, there has been a growing concern that green areas, vegetation, and land-cover characteristics can influence the risk of contracting dengue, as they modify the mosquito population [19]. Dengue vectors can be found in vegetation [20,21]. The spatial distribution of surrounding greenness, mosquitoes, and other insect vectors are interrelated [22,23], since vegetation can provide resting or feeding sites for mosquitoes, or can be a proxy for the presence of breeding sites [24]. However, while previous studies have focused on assessing the land use factors associated with dengue fever, the relationship between green land use factors and DF has not been studied, yet. Furthermore, the relationship between the dengue epidemic and greenness indexes, such as normalized difference vegetation index (NDVI) or enhanced vegetation index (EVI), is not consistent. Some studies indicate that the dengue epidemic reveals a positive association with vegetation [25], while others have found that low vegetation cover areas present increased dengue incidence rates [26,27,28]. This inconsistency may be explained by regional differences.

As there were acute outbreaks of DF in 2014 and 2015 in Taiwan, our study combines geographic information systems with remote sensing, using spatial autocorrelation analysis, and employs two different greenness indexes to understand the influence of different types of green space (such as farm, forest, grassland, and parks) on dengue fever in southern Taiwan.

## 2. Materials and Methods

### 2.1. Study Area

**Figure 1:**
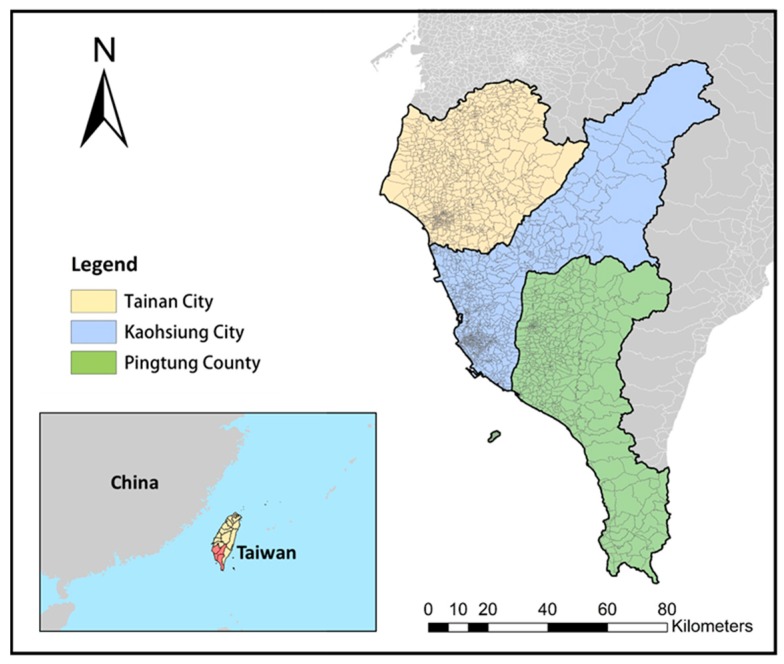
Study area.

Rural and urban areas in Tainan, Kaohsiung, and Pingtung were selected as our study area (Figure 1). All of them are located in southwestern Taiwan, and within the tropical climate zone where *Aedes aegypti* and *Aedes albopictus* can be found. The average temperature ranges from 17 to 29 °C, and annual cumulative precipitation is approximately 1800 mm. The main rainy season is from June to September. In May and June, through the “plum rains” (also known as the East Asian rainy season), the continuous accumulation of rainfall and high humidity are usually brought to this region. Tainan City has an area of 2192 km^2^, with a population of about 1.89 million people. The proportion of land covered with high-tech industrial companies is increasing, but there is still a large area of farming and agriculture land. Kaohsiung City is the largest metropolitan city in southern Taiwan, with a population of 2.9 million and an area of 2952 km^2^. It is also well known as a heavy industrial city, with numerous manufacturing and high-tech industrial areas. Pingtung County covers an area of 2776 km^2^, and has a population of 84 million people, approximately one-third of whom are farmers.

### 2.2. Dengue Data

DF has been classified as notifiable infectious disease category 2 in Taiwan, and suspected cases must be reported within 24 h of clinical diagnosis. Suspected dengue cases are confirmed by the Taiwan Centers for Disease Control (CDC) based on the positive results of a serological test (IgM Enzyme-Linked Immunosorbent Assay), nucleotide sequence, or viral isolation. To monitor and control dengue efficiently, CDC employed the nonstructural protein 1 (NS1) antigen detection as a method of rapid diagnosis since 2014.

For decades, the annual incidence of DF has been the highest among infectious diseases in Taiwan, with cases principally clustered in the southern part of Taiwan. According to the reports from CDC [29], there were 15,394 confirmed autochthonous cases in southern Taiwan in 2014. In 2015, there were 42,932 cases autochthonous reported in southern Taiwan. Information of DF patients between 2014 and 2015 was provided by a computerized database by recording daily notifications of dengue fever cases obtained from the CDC in Taiwan. Details like age, sex, county of residence, and time of disease onset of each patient were included. Our study modeled the incidence rate and number of cases of DF at the village scale. These data serve as a dependent variable in our model.

### 2.3. Greenness Index

Two quantitative metrics were used to evaluate the surrounding greenness in southern Taiwan, including NDVI and green land use area. NDVI information was collected from NASA’s Moderate Resolution Imaging Spectroradiometer (MODIS). NDVI itself varies between −1.0 and +1.0, based on information from visible and infrared red light bands received by satellites, and can be a robust metric for estimating photosynthetically active vegetation coverage. NDVI data are generated every 16 days at 250 m spatial resolution, providing spatial and temporal comparisons of vegetation canopy greenness, the composite properties of leaf area, chlorophyll, and canopy structure [30]. MODIS NDVI Version 6 was applied in this study, which allows a higher temporal resolution, since, in the 16 day period, two 8 day composite reflectance granules (MxD09A1) were used to generate a 16 day composite from. There were two NDVI measures for each cell in every month. Images with the acquisition date closer to mid-month (the fifteenth) were collected and used to calculate the annual average NDVI values for each village.

Green land use area data were obtained from the Taiwan National Land Surveying and Mapping Center (NLSC). These data are vector data, not like NDVI, which is raster data. This digital data layer of land use in Taiwan is based on the 0.25 m resolution digital orthoimagery. The land use data contains 103 different land uses, including agricultural, forest, public utility, residential areas, and so on. Exhaustive descriptions and definitions for every land use are provided on the NLSC website. In this study, we divided green land use into four major categories: farm, forest, park, and grassland. The farm category included paddy fields, wheat fields, and so on. The forest category included combinations of broad-leaf forest, coniferous forest, bamboo forest, and mixed forest. Park was defined as an area of general green amenity space. The grassland category included low vegetation with thick regenerated cover but lacking tall vegetation (trees). Green land use areas within each village were expressed according to area ratio and size, i.e., the percentages and areal sizes of different categories in each village.

### 2.4. Risk Factors

As there are other cofactors that might influence the incidence of DF, risk factors like precipitation, air temperature, population, and income level were added into the model for adjustment. We selected monthly data from 532 meteorological stations and then used kriging estimates to obtain temperature and rainfall data at the village scale in the areas of study. Water body data were obtained from the NLSC, including rivers, reservoirs, lakes, canals, and so on. Population and economic data were gathered from the Fiscal Information Agency of the Ministry of Finance in Taiwan in 2014 and 2015. This dataset comprises the spatial distributions of 12 socioeconomic attributes, such as per capita income, and average population density in each village. Personal economic conditions were expressed based on income tax information. The population densities were derived using the number of people living permanently in the villages in 2014 and 2015, divided by the area (km^2^), which were used to adjust the model and calculate the incidence.

### 2.5. Statistical Analysis

In this study, the Spatial Statistics module of ArcGIS 10.2 software (ESRI Inc., Redlands, CA, USA) was employed to apply spatial autocorrelation analysis. Through Moran’s I, high/low clustering, and hot spot analysis (Hot Spot), we analyze the spatial resolution of the villages in the studied areas in 2014 and 2015, to investigate the spatial agglomeration of dengue fever. Among them, the Moran’s I indicator can indicate whether DF is dispersed or clustered; the high/low analysis can test whether the clustering phenomenon is a high or low value aggregation; lastly, hot point analysis further points out where clustering of hot and cold areas is located.

We use Spearman’s rho to quantify the strength of the relationship between two variables when one or both variables in a correlation analysis is/are not normally distributed. The coefficient of correlation indicates the amount of information common to two variables. This coefficient takes values between −1 and +1, with −1 indicating perfect negative linear association, +1 indicating perfect positive linear association, and 0 indicating no linear relationship.

Generalized linear mixed models (GLMMs) was applied to assess the association between surrounding greenness (represented as NDVI and green land use area) and the dengue epidemic. A negative binomial model was used to explain the overdispersion found in dengue fever count data [31,32]. These models can handle erroneous data that do not follow a normal distribution, and contain random items (grouping variables) to account for temporal or spatial correlation in the data [33,34]. All statistical analyses were performed using the statistical software R (R Foundation for Statistical Computing, Vienna, Austria, version 3.2.2), with the mgcv library.

To confirm the robustness of the associations, sensitivity analysis was performed under two approaches: (1) using the number of cases instead of the incidence rate; and (2) using Kaohsiung City to represent the overall performance of the study area.

Stratified analysis was performed to test the effect on the association between greenness and dengue epidemics in distinct regions, to see if it has the same impact on different areas. Stratification variables include (1) incidence rate of dengue fever in different years, 2014 and 2015, and (2) number of cases in each year.

## 3. Results

### 3.1. Descriptive Statistics

**Figure 2:**
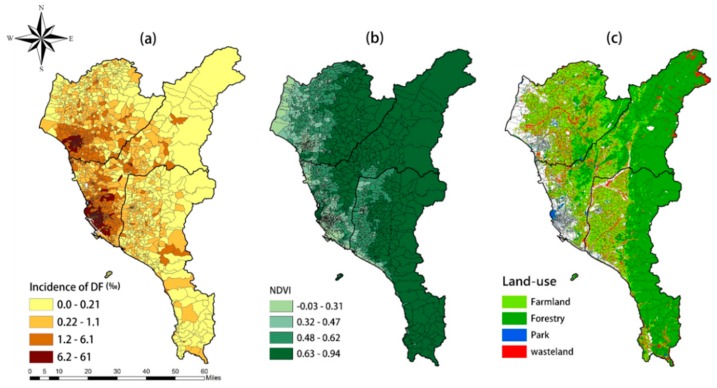
The spatial-temporal analysis of the (**a**) incidence of dengue fever, (**b**) NDVI, and (**c**) green land use.

Figure 2 shows the results of spatial-temporal analysis in southern Taiwan. The dengue epidemic was mainly concentrated in urban areas of Tainan and Kaohsiung, while the distribution in Pingtung was rather scattered. The NDVI in mountainous areas was relatively high, while NDVI in urban areas was generally low. From the results of green land use, most of the farmland is located in lower elevations, and the forests are mostly located at higher altitudes. Park resources are mainly concentrated in urban areas, and grasslands were negatively associated with DF.

On average, Table 1 shows that Pingtung County has the highest percentage of farm, forest, and grassland. Kaohsiung has the highest proportion of recreational area. The ratios of farm and grassland of Tainan City were greater than those of Kaohsiung City, whereas the rate of the forest land was higher in Kaohsiung than in Tainan. Therefore, from our observation, Pingtung had the highest percentage of greenness, followed by Kaohsiung City and, in last place, Tainan City.

### 3.2. Spatial Autocorrelation Analysis

Results of spatial autocorrelation analysis showed a high aggregation of the dengue epidemic in southern Taiwan, and the metropolitan districts of the three studied areas were the main hotspots.

**Figure 3:**
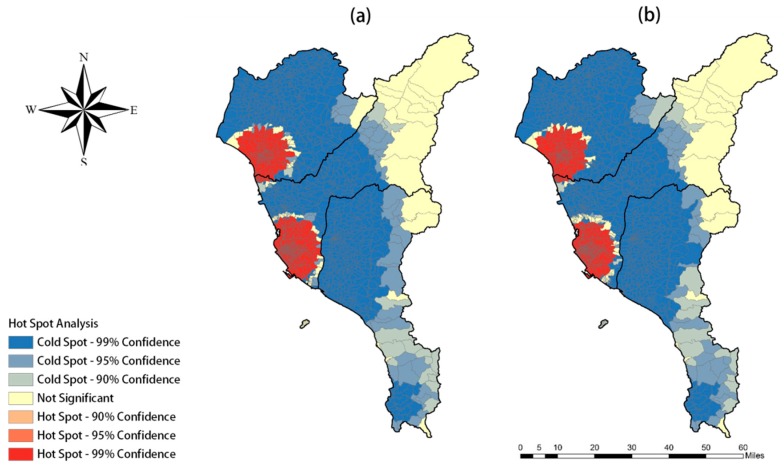
The results of hot spot analysis: (**a**) number of dengue cases; (**b**) dengue fever incidence.

The value of the Moran’s I test for dengue incidence was 0.46, indicating that there was occurrence of spatial clustering in Taiwan. In addition, the result of the high/low aggregation analysis, called Observed General G, and was 0.41, meaning the dengue epidemic was highly aggregated. Finally, based on the result of hot spot analysis (Figure 3), the hot spots of the dengue epidemic were mainly located in urban areas, which is very similar to the result of spatial analysis. All test results were statistically significant (*p* < 0.001).

### 3.3. Spearman’s Rank Correlation Coefficient

In this study, we calculated Spearman’s correlation coefficients between surrounding greenness and the dengue epidemic. We found that there was a significant negative correlation between the incidence of dengue fever and farm, forest and grassland (values between −0.30 to −0.38), however, parks showed a significant, positive correlation with dengue fever (value is 0.21). All green land use (including farms, forest, parks, and grass) and NDVI indicated the overall status of the surrounding greenness in the studied area, and both of them are negatively correlated with the dengue epidemic. That is, when there is lower level of greenness, the dengue fever epidemic becomes more serious.

### 3.4. Generalized Linear Mixed Models

We used GLMM to adjust the social and environmental covariates, which are associated with the incidence rates while considering temporal and spatial correlation (Table 2). There are two types of models, crude models and adjusted models. A crude model examines how a single factor influences the outcome and ignores other socioeconomic covariates. An adjusted model contains potential relevant covariates.

Overall, the results of the model support previous analysis of the relationship between DF and surrounding greenness. The outcomes of the crude model and the adjusted model share the same influence trends, which show significant positive associations among parks and DF, while farm, forest, and grassland were negatively associated with DF, and all analyses reached statistically significant levels (*p* < 0.001).

### 3.5. Sensitivity Test and Stratified Analysis

The outcomes of sensitivity analysis are similar to those of the main model (Table 3), i.e., farm, forest, and grasslands still show significant, negative correlations with dengue fever, while parks show positive correlations.

For the stratified analysis of the number of cases and incident rates of DF in 2014 and 2015 (Table 4), even though some of the results have not achieved significant levels, most of the outcomes show the same trend as the main model, which points out that green land uses like, farm, forest, and grassland, as well as the sum total of greenness area and NDVI, are inversely related to DF, and recreational green areas, like parks, are positively linked with DF.

## 4. Discussion

Many studies have focused on land use factors of dengue vector abundance, yet, few have studied the relationship between green land use factors and DF. Consequently, this study aimed to characterize associations between different types of greenness indexes and the dengue epidemic at the village scale in southern Taiwan, in 2014 and 2015. Spearman’s rho was conducted to reveal the relationship between DF and green land use. Moreover, spatial autocorrelation analysis was used to confirm that dengue fever exhibited a spatial clustering effect in each village, and that hot spots were concentrated in metropolitan areas. Therefore, we further utilized GLMM to adjust the spatial autocorrelation problem.

Risk factors in GLMMs were selected based on the following rationale: First of all, climate factors, like temperature and precipitation, were regarded to be the main contributors to the occurrence of DF [3,26,35]. Temperature can have a considerable impact on mosquito population dynamics [36,37,38]. All stages of the mosquito life cycle, including eggs, immature mosquito development, and survival are partly determined by temperature [39,40,41]. In addition, abundance of the predominant vector, *Aedes aegypti*, is partly regulated by precipitation [42]. Precipitation usually provides water in the container as a breeding site [43]. Several studies have linked poverty or relative poverty to DF, for instance, since poorer areas are characterized by factors that may favor higher DF transmission [29,44,45]. Studies have also revealed that water-related facilities are closely associated with an abundance of *Aedes aegypti* [46,47], particularly in areas with numerous water-holding containers [48], because it provides a suitable habitat for the vector [49].

The results of GLMM and Spearman’s rho were employed to examine the level of contribution of greenness to favorable habitats, which facilitate the life-cycle development of dengue-transmitting mosquitoes, and they share consistent results. According to the results, parks had a significantly positive correlation with the dengue epidemic, while the other five green space metrics and land types were negatively correlated with DF. That is, the more parks in the neighborhood, the higher the dengue fever infection risk among people. While parks are recreational areas where people congregate during their leisure time for recreational uses, such as playing sport, picnicking, relaxing, and so on [50], more people are exposed to mosquitos, leading to a higher biting risk. The increase of biting risk offers opportunities for the mosquitoes to acquire DF by biting an infected person, becoming a virus carrier, and then transmitting the virus to other uninfected individuals [51]. Likewise, those areas with more green spaces (except parks) are mainly located suburban areas in southern Taiwan, where the population density is rather low. Even though some people are infected by dengue virus, since population density is lower, it may be difficult for the virus to transmit and spread. Therefore, negative associations were shown between DF and other green spaces.

Many studies in Taiwan show that the elderly had a higher prevalence rate than those in younger dengue patients, which was in contrast to other Southeast Asian countries [52,53,54]. The length of time that people spend in green spaces may be an important factor that affects incidence of DF. In order to obtain a more precise result, we suggest that future studies conduct surveys to examine personal activity patterns, like the amount of time exposed to greenness within a week, which type of green spaces were involved, are other factors that can be added to the model for further adjustment.

## 5. Conclusions

In this study, many analyses, such as Spearman rho and GLMM, were applied to investigate the relationship between greenness and DF. We hope that our results can serve as a reference for public health departments, and help them to design and implement more effective DF prevention and control measures. As our findings point to a positive correlation between DF incidence and parks, we suggest that control means, such as combined vector control, should focus on park areas.

## Figures and Tables

**Table 1:** The Descriptive Statistics of Different Types of Surrounding Greenness in Southern Taiwan.

Greenness Type	Mean ± Standard Deviation		
	Tainan	Kaohsiung	Pingtung
Farm %	36.2 ± 9.5	17.4 ± 7.0	41.7 ± 9.4
Forest %	8.8 ± 3.9	12.2 ± 7.3	20.3 ± 12.8
Park %	1.5 ± 0.1	4.4 ± 1.1	1.0 ± 0.1
Grass %	3.5 ± 0.3	1.8 ± 0.2	4.2 ± 0.3
All green land use %	52.5 ± 13.3	40.2 ± 16.6	68.7 ± 10.4
NDVI	0.45 ± 0.04	0.41 ± 0.04	0.59 ± 0.03

**Table 2:** Relative Ratios (RR) and Coefficients for Number of Dengue Cases and Different Types of Greenness in Generalized Linear Mixed Models.

Type	Incidence					
	Model ^1^			Model ^2^		
	RR	Coefficients	*p*-Value	RR	Coefficient	*p*-Value
Farm ‰	0.999	−0.001	<0.001	0.999	−0.001	<0.001
Forest ‰	0.999	−0.001	<0.001	0.999	−0.001	<0.001
Park ‰	1.002	0.002	<0.001	1.001	0.001	<0.001
Grass ‰	0.996	−0.004	<0.001	0.997	−0.003	<0.001
All green land use ‰	0.999	−0.001	<0.001	0.999	−0.001	<0.001
NDVI	0.782	−0.246	<0.001	0.998	−0.244	<0.001

^1^ Crude models. ^2^ Adjusted for temperature, rainfall, economy, and bodies of water.

**Table 3:** The Association between Surrounding Greenness and the Dengue Epidemic Kaohsiung and Number of Cases.

Type	Kaohsiung ^1^			Number ^2^		
	RR	Coefficients	*p*-Value	RR	Coefficients	*p*-Value
Farm %	0.998	−0.002	<0.001	0.999	−0.001	<0.001
Forest %	0.998	−0.002	<0.001	0.999	−0.001	<0.001
Park %	1.001	0.001	0.021	1.001	0.001	<0.001
Grass %	0.996	−0.004	<0.001	0.997	−0.003	<0.001
All green land use %	0.999	−0.001	<0.001	0.999	−0.001	<0.001
NDVI	0.802	−0.221	<0.001	0.998	−0.244	<0.001

^1^ Adjusted for temperature, rainfall, economy, and bodies of water. ^2^ Adjusted for temperature, rainfall, economy, bodies of water, and population.

**Table 4:** The Association between Surrounding Greenness and the Dengue Epidemics in 2014 and 2015.

Type	2014						2015					
	Number ^1^			Incidence ^2^			Number ^1^			Incidence ^2^		
	RR	Coefficients	*p*-Value	RR	Coefficients	*p*-Value	RR	Coefficients	*p*-Value	RR	Coefficient	*p*-Value
Farm %	0.978	−0.022	<0.001	0.999	−0.001	<0.001	0.976	−0.024	<0.001	0.998	−0.002	<0.001
Forest %	0.955	−0.046	0.532	0.999	−0.001	<0.001	0.960	−0.041	0.702	1.000	0	0.016
Park %	1.003	0.003	0.478	1.001	0.001	<0.001	1.016	0.016	<0.001	1.001	0.001	0.049
Grass %	0.984	−0.016	0.288	0.999	−0.001	<0.001	0.970	−0.030	<0.001	0.996	−0.004	<0.001
All green land use %	0.980	−0.020	<0.001	0.999	−0.001	<0.001	0.978	−0.022	<0.001	0.999	−0.001	<0.001
NDVI	0.047	−3.052	<0.001	0.913	−0.091	<0.001	0.025	−3.674	<0.001	0.688	−0.374	<0.001

^1^ Adjusted for temperature, rainfall, economy, and bodies of water. ^2^ Adjusted for temperature, rainfall, economy, bodies of water, and population.

## References

[1] Shragai T., Tesla B., Murdock C., Harrington L.C. (2017). Zika and chikungunya: Mosquito-borne viruses in a changing world. Ann. N. Y. Acad. Sci..

[2] Leta S., Beyene T.J., De Clercq E.M., Amenu K., Revie C.W., Kraemer M.U.G. (2018). Global risk mapping for major diseases transmitted by Aedes aegypti and Aedes albopictus. Int. J. Infect. Dis..

[3] Bhatt S., Gething P.W., Brady O.J., Messina J.P., Farlow A.W., Moyes C.L., Drake J.M., Brownstein J.S., Hoen A.G., Sankoh O. (2013). The global distribution and burden of dengue. Nature.

[4] Messina J.P., Brady O.J., Scott T.W., Zou C., Pigott D.M., Duda K.A., Bhatt S., Katzelnick L., Howes R.E., Battle K.E. (2014). Global spread of dengue virus types: Mapping the 70 year history. Trends Microbiol..

[5] Gibbons R.V., Vaughn D.W. (2002). Dengue: An escalating problem. BMJ.

[6] Taylor-Robinson A.W. (2017). Enhancement of Infection by Pre-Existing Non-Neutralizing Antibodies to Cross-Reactive Flaviviruses: Ramifications for Vaccination against Dengue and Zika. J. Vaccines Clin. Trials.

[7] Tomashek K.M., Gregory C.J., Rivera Sánchez A., Bartek M.A., Garcia Rivera E.J., Hunsperger E., Muñoz-Jordán J.L., Sun W. (2012). Dengue Deaths in Puerto Rico: Lessons Learned from the 2007 Epidemic. PLoS Negl. Trop. Dis..

[8] Kakkar M. (2012). Dengue fever is massively under-reported in India, hampering our response. BMJ.

[9] Murray N.E.A., Quam M.B., Wilder-Smith A. (2013). Epidemiology of dengue: Past, present and future prospects. Clin. Epidemiol..

[10] Brady O.J., Gething P.W., Bhatt S., Messina J.P., Brownstein J.S., Hoen A.G., Moyes C.L., Farlow A.W., Scott T.W., Hay S.I. (2012). Refining the Global Spatial Limits of Dengue Virus Transmission by Evidence-Based Consensus. PLoS Negl. Trop. Dis..

[11] Shu P.Y., Su C.L., Liao T.L., Yang C.F., Chang S.F., Lin C.C., Chang M.C., Hu H.C., Huang J.H. (2009). Molecular Characterization of Dengue Viruses Imported Into Taiwan during 2003–2007: Geographic Distribution and Genotype Shift. Am. J. Trop. Med. Hyg..

[12] Kuan M.M., Chang F.Y. (2012). Airport sentinel surveillance and entry quarantine for dengue infections following a fever screening program in Taiwan. BMC Infect. Dis..

[13] Kuan M.M., Lin T., Chuang J.H., Wu H.S. (2010). Epidemiological trends and the effect of airport fever screening on prevention of domestic dengue fever outbreaks in Taiwan, 1998–2007. Int. J. Infect. Dis..

[14] Wu H.H., Wang C.Y., Teng H.J., Lin C., Lu L.C., Jian S.W., Chang N.T., Wen T.H., Wu J.W., Liu D.P. (2013). A dengue vector surveillance by human population-stratified ovitrap survey for Aedes (Diptera: Culicidae) adult and egg collections in high dengue-risk areas of Taiwan. J. Med. Entomol..

[15] Jian Y., Silvestri S., Belluco E., Saltarin A., Chillemi G., Marani M. (2014). Environmental forcing and density-dependent controls of Culex pipiens abundance in a temperate climate (Northeastern Italy). Ecol. Model..

[16] Lebl K., Brugger K., Rubel F. (2013). Predicting Culex pipiens/restuans population dynamics by interval lagged weather data. Parasit. Vectors.

[17] Bhandari K.P., Raju P., Sokhi B.S. (2008). Application of GIS modeling for dengue fever prone area based on socio-cultural and environmental factors—A case study of Delhi city zone. Int. Arch. Photogramm. Remote Sens. Spat. Inf. Sci..

[18] Vanwambeke S.O., van Benthem B.H., Khantikul N., Burghoorn-Maas C., Panart K., Oskam L., Lambin E.F., Somboon P. (2006). Multi-level analyses of spatial and temporal determinants for dengue infection. Int. J. Health Geogr..

[19] Sarfraz M.S., Tripathi N.K., Tipdecho T., Thongbu T., Kerdthong P., Souris M. (2012). Analyzing the spatio-temporal relationship between dengue vector larval density and land-use using factor analysis and spatial ring mapping. BMC Public Health.

[20] Vezzani D., Rubio A., Velázquez S.M., Schweigmann N., Wiegand T. (2005). Detailed assessment of microhabitat suitability for Aedes aegypti (Diptera: Culicidae) in Buenos Aires, Argentina. Acta Tropica.

[21] Hayden M.H., Uejio C.K., Walker K., Ramberg F., Moreno R., Rosales C., Gameros M., Mearns L.O., Zielinski-Gutierrez E., Janes C.R. (2010). Microclimate and human factors in the divergent ecology of Aedes aegypti along the Arizona, U.S./Sonora, MX border. Ecohealth.

[22] Sames W.J., Kim H.C., Chong S.T., Harrison B.A., Won-Ja L., Rueda L.M., Klein T.A. (2008). Anopheles lindesayi japonicus Yamada (Diptera: Culicidae) in Korea: Comprehensive review, new collection records, and description of larval habitats. J. Vector Ecol..

[23] Ferraguti M., la Puente J.M., Roiz D., Ruiz S., Soriguer R., Figuerola J. (2016). Effects of landscape anthropization on mosquito community composition and abundance. Sci. Rep..

[24] Machault V., Vignolles C., Pagès F., Gadiaga L., Gaye A., Sokhna C., Trape J.F., Lacaux J.P., Rogier C. (2010). Spatial heterogeneity and temporal evolution of malaria transmission risk in Dakar, Senegal, according to remotely sensed environmental data. Malar. J..

[25] Meza-Ballesta A., Gónima L. (2014). The influence of climate and vegetation cover on the occurrence of dengue cases (2001–2010). Rev. Salud-Publica. (Bogota).

[26] Troyo A., Fuller D.O., Calderón-Arguedas O., Solano M.E., Beier J.C. (2009). Urban structure and dengue fever in Puntarenas, Costa Rica. Singap. J. Trop. Geogr..

[27] Araujo R.V., Albertini M.R., Costa-da-Silva A.L., Suesdek L., Franceschi N.C.S., Bastos N.M., Katz G., Cardoso V.A., Castro B.C., Capurro M.L. (2015). São Paulo urban heat islands have a higher incidence of dengue than other urban areas. Braz. J. Infect. Dis..

[28] Qi X., Wang Y., Li Y., Meng Y., Chen Q., Ma J., Gao G.F. (2015). The Effects of Socioeconomic and Environmental Factors on the Incidence of Dengue Fever in the Pearl River Delta, China, 2013. PLoS Negl. Trop. Dis..

[29] Center for Disease Control (2016). Taiwan National Infectious Disease Statistics System for Dengue Virus Surveillance. http://nidss.cdc.gov.tw/en/SingleDisease.aspx?dc=1&dt=2&disease=061.

[30] Gascon M., Cirach M., Martínez D., Dadvand P., Valentín A., Plasència A., Nieuwenhuijsen M.J. (2016). Normalized difference vegetation index (NDVI) as a marker of surrounding greenness in epidemiological studies: The case of Barcelona city. Urban Forest. Urban Green..

[31] Zellweger R.M., Cano J., Mangeas M., Taglioni F., Mercier A., Despinoy M., Menkès C.E., Dupont-Rouzeyrol M., Nikolay B., Teurlai M. (2017). Socioeconomic and environmental determinants of dengue transmission in an urban setting: An ecological study in Nouméa, New Caledonia. PLoS Negl. Trop. Dis..

[32] Fairos W.Y.W., Azaki W.H.W., Alias L.M., Wah Y.B. (2010). Modelling dengue fever (DF) and dengue haemorrhagic fever (DHF) outbreak using Poisson and Negative Binomial model. World Acad. Sci. Eng. Technol..

[33] Paterson S., Lello J. (2003). Mixed models: Getting the best use of parasitological data. Trends Parasitol..

[34] Mohebbi M., Wolfe R., Jolley D. (2011). A poisson regression approach for modelling spatial autocorrelation between geographically referenced observations. BMC Med. Res. Methodol..

[35] Morin C.W., Comrie A.C. (2010). Modeled response of the West Nile virus vector Culex quinquefasciatus to changing climate using the dynamic mosquito simulation model. Int. J. Biometeorol..

[36] Scott T.W., Amerasinghe P.H., Morrison A.C., Lorenz L.H., Clark G.G., Strickman D., Kittayapong P., Edman J.D. (2000). Longitudinal studies of Aedes aegypti (Diptera: Culicidae) in Thailand and Puerto Rico: Blood feeding frequency. J. Med. Entomol..

[37] Wilke A.B.B., Medeiros-Sousa A.R., Ceretti-Junior W., Marrelli M.T. (2017). Mosquito populations dynamics associated with climate variations. Acta Tropica.

[38] Ewing D.A., Cobbold C.A., Purse B.V., Nunn M.A., White S.M. (2016). Modelling the effect of temperature on the seasonal population dynamics of temperate mosquitoes. J. Theor. Biol..

[39] Focks D.A., Haile D.G., Daniels E., Mount G.A. (1993). Dynamic Life Table Model for Aedes aegypti (Diptera: Culicidae): Analysis of the Literature and Model Development. J. Med. Entomol..

[40] Ciota A.T., Matacchiero A.C., Kilpatrick A.M., Kramer L.D. (2014). The Effect of Temperature on Life History Traits of Culex Mosquitoes. J. Med. Entomol..

[41] Madder D.J., Surgeoner G.A., Helson B.V. (1983). Number of Generations, Egg Production, and Developmental Time of Culex Pipiens and Culex Restuans (Diptera: Culicidae) in Southern Ontario. J. Med. Entomol..

[42] Morin C.W., Comrie A.C., Ernst K. (2013). Climate and dengue transmission: Evidence and implications. Environ. Health Perspect..

[43] Sirisena P., Noordeen F., Kurukulasuriya H., Romesh T.A., Fernando L. (2017). Effect of Climatic Factors and Population Density on the Distribution of Dengue in Sri Lanka: A GIS Based Evaluation for Prediction of Outbreaks. PLoS ONE.

[44] Costa J.V., Donalisio M.R., Silveira L.V. (2013). Spatial distribution of dengue incidence and socio-environmental conditions in Campinas, São Paulo State, Brazil, 2007. Cad. Saude Publica.

[45] Mena N., Troyo A., Bonilla-Carrión R., Calderón-Arguedas O. (2011). Factors associated with incidence of dengue in Costa Rica. Rev. Panam. Salud Publica.

[46] Tuan Y.C., Hung M.N., Lin L.J., Shih W.Y., Huang C.C., Chang C., Chen M.J., You C.Y. (2009). Analysis on dengue vector density survey in Kaohsiung and Pingtung areas of southern Taiwan, 2004–2008. Epidemiol. Bull..

[47] Saifur R.G.M., Hassan A.A., Dieng H., Salmah M.R.C., Saad A.R., Satho T. (2013). Temporal and spatial distribution of dengue vector mosquitoes and their habitat patterns in Penang Island, Malaysia. J. Am. Mosq. Control Assoc..

[48] Shang C.S., Fang C.T., Liu C.M., Wen T.H., Tsai K.H., King C.C. (2010). The Role of Imported Cases and Favorable Meteorological Conditions in the Onset of Dengue Epidemics. PLoS Negl. Trop. Dis..

[49] Cheong Y.L., Leitão P.J., Lakes T. (2014). Assessment of land use factors associated with dengue cases in Malaysia using Boosted Regression Trees. Spat. Spatio. Temporal. Epidemiol..

[50] Kruger J. (2008). Parks, Recreation, and Public Health Collaborative. Environ. Health Insights.

[51] Scott T.W., Morrison A.C. (2010). Longitudinal Field Studies Will Guide a Paradigm Shift in Dengue Prevention. Vector Biology, Ecology and Control.

[52] Hsu J.C., Hsieh C.L., Lu C.Y. (2017). Trend and geographic analysis of the prevalence of dengue in Taiwan, 2010–2015. Int. J. Infect. Dis..

[53] Yeh C.Y., Chen P.L., Chuang K.T., Shu Y.C., Chien Y.W., Perng G.C., Ko W.C., Ko N.Y. (2017). Symptoms associated with adverse dengue fever prognoses at the time of reporting in the 2015 dengue outbreak in Taiwan. PLoS Negl. Trop. Dis..

[54] Chuang T.W., Ng K.C., Nguyen T.L., Chaves L.F. (2018). Epidemiological Characteristics and Space-Time Analysis of the 2015 Dengue Outbreak in the Metropolitan Region of Tainan City, Taiwan. Int. J. Environ. Res. Public Health.

